# Transport injuries and deaths in the Eastern Mediterranean Region: findings from the Global Burden of Disease 2015 Study

**DOI:** 10.1007/s00038-017-0987-0

**Published:** 2017-08-03

**Authors:** GBD 2015 Eastern Mediterranean Region Transportation Injuries Collaborators, Ibrahim Khalil, Charbel El Bcheraoui, Raghid Charara, Maziar Moradi-Lakeh, Ashkan Afshin, Nicholas J. Kassebaum, Michael Collison, Farah Daoud, Adrienne Chew, Kristopher J. Krohn, Danny Colombara, Leslie Cornaby, Rebecca Ehrenkranz, Nicholas Graetz, Michael Kutz, Christopher Troeger, Haidong Wang, Kalkidan Hassen Abate, Foad Abd-Allah, Abdishakur M. Abdulle, Semaw Ferede Abera, Aliasghar Ahmad Kiadaliri, Alireza Ahmadi, Muktar Beshir Ahmed, Khurshid Alam, Deena Alasfoor, Suliman Alghnam, Raghib Ali, Reza Alizadeh-Navaei, Rajaa Al-Raddadi, Ubai Alsharif, Khalid A. Altirkawi, Nahla Anber, Hossein Ansari, Carl Abelardo T. Antonio, Palwasha Anwari, Hamid Asayesh, Tesfay Mehari Atey, Leticia Avila-Burgos, Suzanne L. Barker-Collo, Shahrzad Bazargan-Hejazi, Neeraj Bedi, Addisu Shunu Beyene, Zulfiqar A. Bhutta, Soufiane Boufous, Zahid A. Butt, Carlos A. Castañeda-Orjuela, Abdulaal A. Chitheer, Koustuv Dalal, Hadi Danawi, Dragos V. Davitoiu, Shirin Djalalinia, Aman Yesuf Endries, Babak Eshrati, Alireza Esteghamati, André Faro, Maryam S. Farvid, Seyed-Mohammad Fereshtehnejad, Florian Fischer, Wayne Gao, Solomon Weldemariam Gebrehiwot, Tsegaye Tewelde Gebrehiwot, Nima Hafezi-Nejad, Hassan Haghparast Bidgoli, Gessessew Bugssa Hailu, Randah Ribhi Hamadeh, Samer Hamidi, Delia Hendrie, Ileana Beatriz Heredia-Pi, Kathryn H. Jacobsen, Spencer Lewis James, Achala Upendra Jayatilleke, Guohong Jiang, Jost B. Jonas, Amir Kasaeian, Peter Njenga Keiyoro, Yousef Saleh Khader, Ejaz Ahmad Khan, Abdullah Tawfih Abdullah Khoja, Ardeshir Khosravi, Jagdish Khubchandani, Yun Jin Kim, Soewarta Kosen, Barthelemy Kuate Defo, Heidi J. Larson, Shai Linn, Raimundas Lunevicius, Hassan Magdy Abd El Razek, Mohammed Magdy Abd El Razek, Marek Majdan, Reza Majdzadeh, Azeem Majeed, Reza Malekzadeh, Peter Memiah, Ziad A. Memish, Walter Mendoza, Mubarek Abera Mengistie, Tuomo J. Meretoja, Ted R. Miller, Shafiu Mohammed, Ashagre Molla Assaye, Carla Makhlouf Obermeyer, Farshad Pourmalek, Mostafa Qorbani, Amir Radfar, Anwar Rafay, Vafa Rahimi-Movaghar, Mahfuzar Rahman, Rajesh Kumar Rai, Kavitha Ranganathan, David Laith Rawaf, Salman Rawaf, Amany H. Refaat, Andre M. N. N. Renzaho, Satar Rezaei, David Rojas-Rueda, Gholamreza Roshandel, Mahdi Safdarian, Saeid Safiri, Mohammad Ali Sahraian, Payman Salamati, Abdallah M. Samy, Juan Ramon Sanabria, Milena M. Santric Milicevic, Benn Sartorius, David C. Schwebel, Sadaf G. Sepanlou, Amira Shaheen, Masood Ali Shaikh, Mansour Shamsipour, Morteza Shamsizadeh, Badr H. A. Sobaih, Muawiyyah Babale Sufiyan, Jacob E. Sunshine, Arash Tehrani-Banihashemi, Mohamad-Hani Temsah, Abdullah Sulieman Terkawi, J. S. Thakur, Roman Topor-Madry, Olalekan A. Uthman, Vasiliy Victorovich Vlassov, Stein Emil Vollset, Tolassa Wakayo, Andrea Werdecker, Mohsen Yaghoubi, Mehdi Yaseri, Naohiro Yonemoto, Mustafa Z. Younis, Maysaa El Sayed Zaki, Aisha O. Jumaan, Theo Vos, Mohsen Naghavi, Simon I. Hay, Christopher J. L. Murray, Ali H. Mokdad

**Affiliations:** Institute for Health Metrics and Evaluation, 2301 5th Avenue, Suite 600, Seattle, WA 98121 USA

**Keywords:** Transport injuries, Eastern Mediterranean Region, Burden of disease

## Abstract

**Objectives:**

Transport injuries (TI) are ranked as one of the leading causes of death, disability, and property loss worldwide. This paper provides an overview of the burden of TI in the Eastern Mediterranean Region (EMR) by age and sex from 1990 to 2015.

**Methods:**

Transport injuries mortality in the EMR was estimated using the Global Burden of Disease mortality database, with corrections for ill-defined causes of death, using the cause of death ensemble modeling tool. Morbidity estimation was based on inpatient and outpatient datasets, 26 cause-of-injury and 47 nature-of-injury categories.

**Results:**

In 2015, 152,855 (95% uncertainty interval: 137,900–168,100) people died from TI in the EMR countries. Between 1990 and 2015, the years of life lost (YLL) rate per 100,000 due to TI decreased by 15.5%, while the years lived with disability (YLD) rate decreased by 10%, and the age-standardized disability-adjusted life years (DALYs) rate decreased by 16%.

**Conclusions:**

Although the burden of TI mortality and morbidity decreased over the last two decades, there is still a considerable burden that needs to be addressed by increasing awareness, enforcing laws, and improving road conditions.

**Electronic supplementary material:**

The online version of this article (doi:10.1007/s00038-017-0987-0) contains supplementary material, which is available to authorized users.

## Introduction

Transport injuries (TI) are a major cause of global mortality and morbidity. In 2015, they caused 1.5 million deaths globally [95% Uncertainty Interval (UI) 1.4–1.5 million] (Wang et al. 2016). In addition to deaths on the roads, up to 50 million people incur nonfatal injuries each year as a result of road traffic crashes and other accidents (GBD 2015 Disease and Injury Incidence and Prevalence Collaborators 2016). The significance of this public health threat is most pronounced in low- and middle-income countries (LMIC), where 90% of the world’s road traffic-related deaths take place. It is projected to be the fifth leading cause of mortality around the world through the year 2030 (Naeem 2010). Transport injuries also exert a significant impact on the affected families, health care services, and national economies (Ainy et al. 2014). Moreover, TI are estimated to cause approximately 3% loss of gross domestic product (GDP) in LMIC (WHO 2015).

The causes of TI can be attributed to different factors: excessive speed, consumption of drugs and alcohol, failure to enforce the use of protective measures such as seatbelts and helmets, poor vehicle impact protection, and poor road conditions (Keay and Simmonds 2005). The road user, the vehicle, and the built environment are elements of a dynamic system that work together to either produce or prevent injuries. Many factors can also influence the frequency and nature of road crashes, including weather conditions, school holidays, time of the day, and alcohol consumption (Sukhai et al. 2011; Karacasu et al. 2011).

The Eastern Mediterranean Region (EMR) contains over 600 million people and consists of 22 countries with varying levels of national income: Afghanistan, Arab Republic of Egypt, Bahrain, Djibouti, Iraq, Islamic Republic of Iran, Jordan, Kingdom of Saudi Arabia (KSA), Kuwait, Lebanon, Libya, Morocco, Oman, Pakistan, Palestine, Qatar, Republic of Yemen, Somalia, Sudan, Syrian Arab Republic (Syria), Tunisia, and the United Arab Emirates (UAE). Although the overall number of registered vehicles per 1000 population is comparatively low (96 per 1000 population), the case-fatality rate from TI is one of the highest in the world (WHO Regional Office for the Eastern Mediterranean 2017). Despite this, studies surrounding this topic are scarce, and reliable data are limited. In addition to their fatality burden, road traffic crashes also increase the burden of nonfatal injuries (Chandran et al. 2010). According to the World Health Organization (WHO) estimates, TI were ranked as the sixth leading cause of death in the EMR, surpassing tuberculosis, malaria, and HIV/AIDS, and the region has the second highest road traffic fatality rate in the world (Kassebaum et al. 2016). In this manuscript, we assessed the burden of TI in the EMR by age and sex from 1990 to 2015, and compared the burden to the global TI, from the Global Burden of Diseases, Injuries, and Risk Factors Study 2015.

## Methods

Transport injuries estimates included pedestrian, cyclist, motorcyclist, and motor vehicle road injuries, in addition to other water and air transport injuries. GBD 2015 estimated injury mortality from vital registration, verbal autopsy, mortality surveillance, censuses, surveys, and police record data. Police and crime reports were used as data sources only for the estimation of deaths from road traffic injuries (Wang et al. 2016). The police data were collected from published studies, national agencies, and institutional surveys such as the United Nations Crime Trends Survey and the WHO Global Status Report on Road Safety Survey. For countries with vital registration data we did not use police records, except if the recorded number of road injury deaths from police records exceeded that in the vital registration.

We assessed mortality by mapping all data sources to the GBD cause list of diseases and injuries, and then adjustments were made for ill-defined causes of death, or garbage codes. Finally, ensemble models with varying choices of covariates and mathematical models were run using the GBD Cause of Death Ensemble modeling (CODEm) software to derive estimates by age, sex, country, year, and cause. Final fatal discontinuity estimations for these causes were merged with CODEm results post‐Cause of Death Correct (CoD Correct) to produce final cause of death results. CoD Correct is a process that uses a simple algorithm to scale all cause-specific deaths from all causes for each age group, sex, year, and location, and thereby ensures that the sum equals total all-cause mortality (Wang et al. 2016).

The preparation of cause of death data, the redistribution of garbage codes, the modeling process, and covariates are explained in more detail elsewhere (Wang et al. 2016). The International Classification of Diseases (ICD) was used to classify injuries. In GBD 2015, injury incidence and deaths are defined as ICD‐9 codes E000‐E999 and ICD‐10 chapters V–Y. More details can be found in a full description of GBD 2015 study methodology (Wang et al. 2016).

We estimated incidence of injury warranting inpatient admission (“inpatient care”) and incidence of injury warranting other types of care (“outpatient care”) for all cause-of-injury categories. Injuries warranting inpatient care refer to injury cases of sufficient severity to require inpatient care assuming no restrictions in access to health care. More details about data sources and our strategy to assess the nonfatal burden of disease can be found elsewhere (Kassebaum et al. 2016).

We calculated years of life lost (YLLs) by multiplying deaths by the residual expected individual life span at the age of death as derived from the GBD 2015 standard model life table (Wang et al. 2016). Years lived with disability (YLDs) were calculated by multiplying the number of prevalent cases of a certain health outcome by the disability weight assigned to this health outcome. A disability weight reflects the magnitude of the health loss associated with an outcome and has a value that is anchored between 0, equivalent to full health, and 1, equivalent to death. Disability-adjusted life years (DALYs) were calculated by adding YLLs and YLDs.

We evaluated the associations between TI and socio-demographic status using the Socio-demographic Index (SDI). SDI is a composite measure developed for GBD 2015 that accounts for fertility rate, lag-dependent income per capita, and education (Wang et al. 2016). To capture the average relationships for each age–sex group, we applied a simple least squares spline regression of the TI mortality rate on SDI. The SDI is scaled from 0 to 1, where 0 represents the lowest possible observed SDI and 1 is the highest. We reported uncertainty for all our estimates (Kassebaum et al. 2016), which have varying degrees of uncertainty arising from input data, the data adjustments and the statistical models. We have propagated uncertainty from all these sources using standard GBD methods of repeating all calculations 1000 times, each time drawing from distributions rather than point estimates for all the relevant parameters in our models (Kassebaum et al. 2016). For the injury mortality estimates the estimation of model uncertainty is inherent to the ensemble modeling method (Wang et al. 2016).

## Results

### Mortality

In 2015, there were 152,855 deaths due to TI in EMR (UI 137,873–168,097), and 1,466,557 deaths globally (UI 1,394,757–1,536,454). In 2015, TI was the eighth leading cause of death in EMR, but the second leading cause of death in Qatar, Oman, and UAE. The percentage of deaths of TI out of total deaths was the highest in Qatar (20%), Oman (16%), and UAE (14%), and the lowest in Pakistan (1.9%), Lebanon (1.8%), and Somalia (1.5%). TI accounted for 27.8 (UI 25.1–30.5) deaths per 100,000 population, higher than the global mortality rate of 20.24 (UI 9.3–21.2) per 100,000 population (Wang et al. 2016). For the region, TI accounted for 3% of all deaths.

**Fig. 1:**
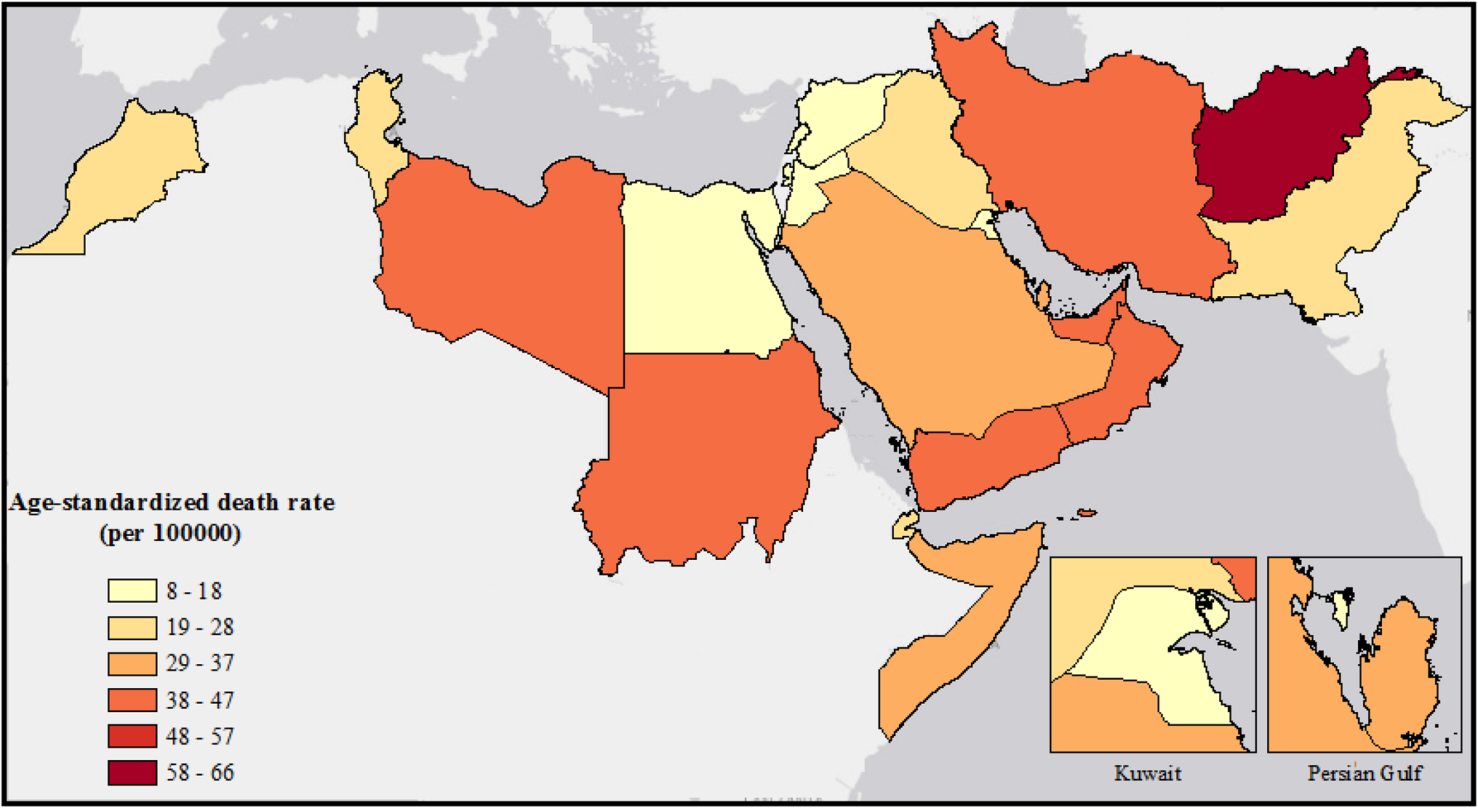
Map of age-standardized mortality rates for transport injuries in the Eastern Mediterranean Region, both sexes, in 2015. (Global Burden of Disease Study 2015, Eastern Mediterranean Countries, 2015)

**Fig. 2:**
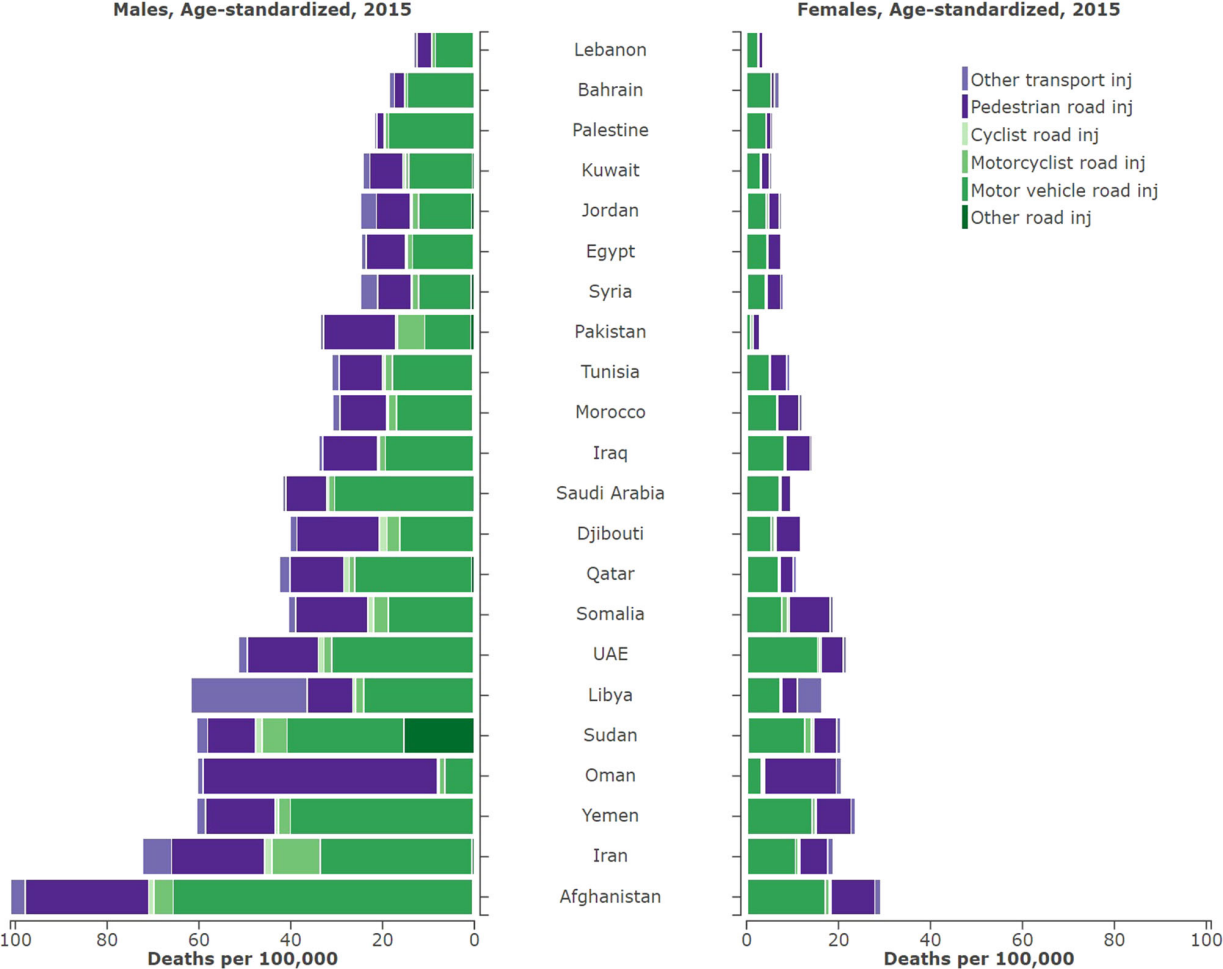
Age-standardized mortality rates for sub-causes of transport injuries in the Eastern Mediterranean Region, by sex and by country, in 2015. (Global Burden of Disease Study 2015, Eastern Mediterranean Countries, 2015)

In 2015, Afghanistan had the highest mortality rate in the region at 66.2 (UI 48.7–87.9) and Lebanon had the lowest at 8.5 (UI 5.7–12.7) per 100,000 population (Fig. 1; Table 1). From 1990 to 2015 Libya, Pakistan, and Egypt had 37, 14.8, and 12.4% increases in TI mortality rate, respectively (Table 1). Three countries, UAE, Kuwait, and Qatar, have a high SDI score, with TI mortality rates significantly higher in UAE (36.8 per 100,000) and Qatar (29.99 per 100,000) than the global average for high-income countries (12.2 per 100,000). Motor vehicle road injuries and pedestrian road injuries were the leading causes for age-standardized mortality rates in the region in 2015 (Fig. 2).

**Table 1:** Age-standardized death rates, YLLs, YLDs, and DALYs for transport injuries in the Eastern Mediterranean Region by country, in 2015

Country	Age standardized death rates (per 100,000)			Age-standardized YLL rate 2015 (per 100,000)	Age-standardized YLD rate 2015 (per 100,000)	Age-standardized DALY rate 2015 (per 100,000)	YLL/YLD ratio	
	1990	2015	% Change				1990	2015
Eastern Mediterranean Region	29.5 (26.4–32.5)	27.8 (25.1–30.5)	−5.8	1187.5 (1072.8–1308.1)	61.4 (43.2–82.8)	1248.9 (1131–1375.1)	20.59	19.34
Afghanistan	70.8 (50.9–91.6)	66.2 (48.7–87.9)	−6.5	3090.3 (2324.6–4115.3)	61.4 (43.9–81.6)	3151.7 (2378–4188.6)	58.71	50.36
Bahrain	28.5 (24.9–33.1)	14 (11.5–17.2)	−50.9	550.5 (449.6–682.7)	68.8 (47.3–94.3)	619.3 (515.8–757.3)	10.48	8.00
Djibouti	27 (16.6–42.6)	25.8 (12.9–48.4)	−4.6	976.1 (490.6–1906.3)	42.7 (30.5–57.2)	1018.8 (530.8–1949.4)	24.83	22.86
Egypt	14.3 (13.1–16.6)	16.1 (14.9–17.5)	12.4	693.7 (634.4–755.7)	45.5 (31.9–61.8)	739.1 (678.2–800.6)	12.01	15.26
Iran	53.7 (45–64.3)	46 (36.7–57.3)	−14.5	1884.9 (1488.9–2375.3)	96.9 (67.4–132.5)	1981.8 (1588.3–2478.4)	24.23	19.45
Iraq	29.2 (23.2–37.3)	24.1 (18–31.7)	−17.5	1068.4 (791.3–1418.2)	65 (46.3–87.6)	1133.4 (856.4–1486.9)	18.56	16.43
Jordan	29.5 (23.3–35.8)	16.3 (14.2–18.7)	−44.7	772.6 (667.3–883.6)	54.3 (37.9–74.6)	826.9 (718.9–940.6)	14.64	14.22
Kuwait	23.1 (21.6–24.8)	16.1 (13.7–19.3)	−30.4	620.5 (529.2–744.2)	82.7 (57–113.2)	703.1 (602.2–828.2)	8.03	7.50
Lebanon	17.1 (13.6–21.6)	8.5 (5.7–12.7)	−50.4	372.1 (248.8–564)	57.7 (40.1–78.9)	429.7 (306.3–620.3)	9.89	6.45
Libya	28.3 (23.2–33.8)	38.8 (28.8–48.6)	37.0	1724.6 (1275.5–2169.5)	75.4 (52–103.1)	1800.1 (1347.9–2247.5)	13.16	22.87
Morocco	29.9 (24.5–35.6)	21.1 (16–28)	−29.3	901.3 (682.2–1194.6)	65.8 (47–88.3)	967.1 (745.4–1261.2)	18.10	13.70
Oman	70.9 (51.4–91.7)	46 (37.7–56)	−35.0	1851.1 (1537.6–2256.4)	116.2 (80.3–159)	1967.4 (1641.1–2371.3)	22.73	15.93
Pakistan	16.2 (12.8–21.4)	18.6 (13.4–25)	14.8	718.5 (525.4–959.3)	36.1 (25.9–48.1)	754.6 (563–1003.5)	18.48	19.89
Palestine	17.1 (13.4–21.7)	13.7 (10.7–17.5)	−19.6	675.8 (521.5–871.5)	44.2 (30.4–60.6)	720 (565.3–917.4)	15.36	15.29
Qatar	53.6 (45.7–62)	33.5 (25.1–42.9)	−37.5	1335 (1024.1–1693.9)	120.3 (82.4–164.5)	1455.3 (1136.6–1808.1)	11.54	11.09
Saudi Arabia	40.5 (33.5–46.3)	27.9 (24–31.7)	−31.1	1125.1 (987–1269.2)	85.7 (58.9–117.6)	1210.7 (1061.2–1355.5)	15.24	13.13
Somalia	31.1 (10.6–66.1)	29.3 (10.1–66)	−5.9	1111.3 (401.5–2630.2)	29.8 (21.5–39.8)	1141 (429.8–2657.2)	40.08	37.31
Sudan	45.6 (31–68.2)	40.2 (27.1–58.6)	−11.9	1957.7 (1298.2–2886.1)	84.6 (60.2–113.5)	2042.4 (1384.1–2978.8)	32.74	23.13
Syria	23.5 (18.4–28)	16.3 (13.1–18.8)	−30.7	640.6 (529.8–741.2)	50.6 (35.3–68.8)	691.2 (576–791.7)	11.97	12.67
Tunisia	30.5 (26–35.5)	19.8 (15.7 to 24.7)	−35.0	756.3 (604.9–956)	61.3 (42.3–84.3)	817.7 (665.2–1021.6)	15.48	12.33
United Arab Emirates	61.3 (43.3–78.7)	43.1 (31.5 to 55.4)	−29.7	1533 (1114.5–1976.1)	119.4 (82.1–163.2)	1652.4 (1233–2087.7)	12.32	12.84
Yemen	46.6 (25.2–74.1)	42 (24.4–68.7)	−10.0	1945.3 (1166.7–3104.7)	76 (54.7–101.5)	2021.3 (1245.1–3175.3)	33.17	25.59

*DALY* disability-adjusted life-years, *YLD* years lived with disability, *YLL* years of life lost. (Global Burden of Disease Study 2015, Eastern Mediterranean Countries, 1990–2015)

Males were substantially more affected than females, with an overall mortality rate of 43.6 (UI 38.5–48.5) per 100,000, compared to a rate of 11.36 (UI 10.1–12.9) per 100,000 for females (Fig. 2). The ratio of age-standardized TI mortality between males and females in the EMR was 3.84 compared to 3.1 globally. Within the EMR, Pakistan had, by far, the highest ratio of mortality in males to females at 12.0, with the next being Kuwait at 4.5.

Table 2 shows observed-to-expected (based exclusively on SDI) ratios for sub-causes of TI by country in 2015. The observed-to-expected ratio varied substantially across both country and sub-cause. The rate of pedestrian injuries in Oman was nearly four times higher than expected. Pedestrian injuries in Oman had a ratio of 4.9, an observed 37.9 (UI 30.7–46.3) deaths per 100,000 compared to an expected 7.66. Only in Oman were pedestrian injuries the largest sub-cause of TI (Fig. 2). Afghanistan, Iran, and Qatar all had observed values for multiple sub-causes that greatly exceeded the expected values (Table 2).

**Table 2:** Ratio of observed mortality rates to expected mortality rates on the basis of SDI alone for sub-causes of transport injuries in the Eastern Mediterranean Region, by country, in 2015. (Global Burden of Disease Study 2015, Eastern Mediterranean Countries, 2015)

Country	Observed/expected age-standardized death rates for transport injuries 2015					
	Pedestrian	Cyclist	Motorcyclist	Motor vehicle	Other road injuries	Other transport injuries
Afghanistan	1.319	1.005	*0.918*	***3.511***	***2.151***	***1.678***
Bahrain	**0.265**	**0.095**	**0.150**	1.156	*0.682*	*0.985*
Djibouti	*0.683*	1.192	**0.499**	*0.878*	*0.744*	*0.561*
Egypt	**0.403**	**0.244**	**0.172**	*0.721*	**0.498**	*0.501*
Iran	***1.693***	*0.975*	***2.088***	***2.202***	***1.948***	***3.107***
Iraq	*0.524*	**0.264**	**0.210**	1.073	*0.687*	**0.471**
Jordan	*0.555*	**0.195**	**0.319**	*0.751*	*0.611*	***1.567***
Kuwait	***1.995***	*0.562*	**0.263**	***2.115***	***1.651***	1.402
Lebanon	**0.324**	**0.126**	**0.168**	*0.610*	**0.433**	**0.465**
Libya	*0.561*	**0.360**	**0.318**	1.340	*0.849*	***10.873***
Morocco	**0.436**	**0.237**	**0.278**	*0.931*	*0.598*	*0.683*
Oman	***4.955***	**0.321**	**0.295**	**0.483**	***1.938***	*0.897*
Pakistan	*0.502*	**0.294**	*0.929*	**0.453**	*0.533*	**0.260**
Palestine	**0.077**	**0.154**	**0.118**	*0.892*	**0.387**	**0.286**
Qatar	***1.905***	0.828	**0.333**	***2.402***	***1.851***	***1.539***
Saudi Arabia	*0.937*	**0.309**	**0.319**	***2.228***	1.434	*0.510*
Somalia	1.040	1.245	*0.891*	1.060	1.036	1.013
Sudan	**0.467**	1.129	1.036	***1.646***	1.197	*0.971*
Syria	**0.309**	**0.202**	**0.214**	*0.604*	**0.414**	1.342
Tunisia	*0.584*	**0.331**	**0.285**	*0.977*	*0.707*	*0.765*
United Arab Emirates	***5.292***	1.399	*0.725*	***5.881***	***4.362***	***1.759***
Yemen	*0.705*	*0.543*	*0.505*	***2.373***	1.278	*0.911*

### Years of life lost (YLLs)

**Fig. 3:**
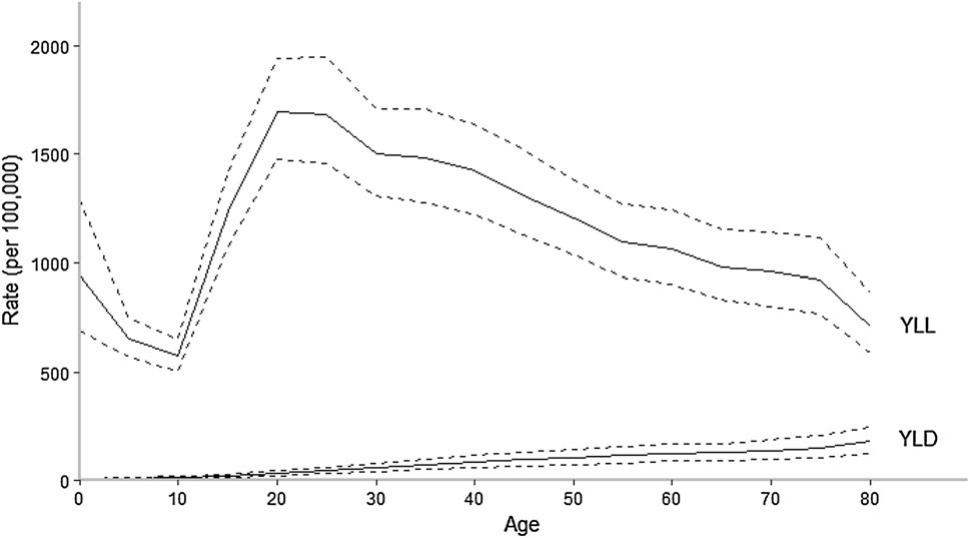
YLL and YLD rates for transport injuries in the Eastern Mediterranean Region, both sexes, in 2015. *YLD* years lived with disability, *YLL* years of life lost. (Global Burden of Disease Study 2015, Eastern Mediterranean Countries, 2015)

The rate of age-adjusted YLLs per 100,000 population was significantly higher in the EMR than globally, 1187.5 (UI 1072.8–1308.1) compared to 881.2 (UI 837.6–923.2) per 100,000 population (Table 1). By age, YLLs peaked in the 20–24 age group, and then steadily decreased as age increased (Fig. 3). This was consistent with the global trend. By country, Afghanistan had the highest age-standardized YLL rate at 3090.3 (UI 2324.6–4115.3) per 100,000 population, nearly three times the regional average (Table 1).

### Years lived with disability (YLDs)

Unlike YLLs, the rate of age-adjusted YLDs in the EMR was lower than the global average, 61.4 (UI 43.2–82.8) compared to 90.2 (UI 63.0–122.2) (Wang et al. 2016). The percentage of YLDs attributable to TI was also lower in the EMR than globally. YLDs steadily increased with age (Fig. 3). Oman, Qatar, and the UAE had the highest age-standardized YLD rates at 116.2, 120.3, and 119.4, respectively (Table 1). Somalia had the lowest rate, at 29.8 (UI 21.5–39.8) (Table 1). In all countries, motor vehicle injuries were the leading sub-cause of YLDs (Table 1).

The YLL/YLD ratio in the EMR for 2015 was 19.34, almost double the global ratio of 9.77 (Wang et al. 2016). By country, Afghanistan had the highest ratio at 50.36, and only Kuwait and Lebanon were below the global ratio at 7.50 and 6.45, respectively (Table 1). For all age groups and countries, YLLs were the primary contributor to DALYs in terms of TI.

### Disability-adjusted life years (DALYs)

**Fig. 4:**
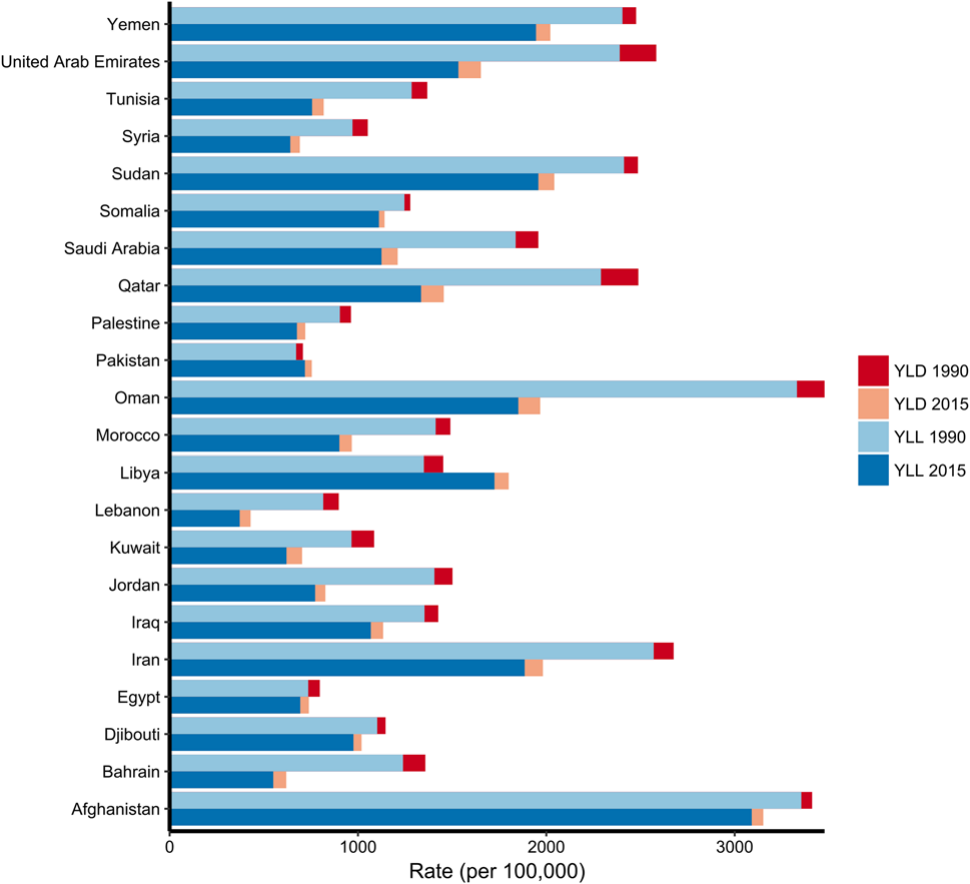
DALY rates per 100,000 population for transport injuries in the Eastern Mediterranean Region both sexes combined, by country, in 1990 and 2015. *DALY* disability-adjusted life-years, *YLD* years lived with disability, *YLL* years of life lost. (Global Burden of Disease Study 2015, Eastern Mediterranean Countries, 1990–2015)

In 2015, TI were the eleventh leading cause DALYs, causing 8,069,712 (95% UI 7,303,759–8,888,094) DALYs. The highest and lowest age-standardized rates of DALYs were observed in Afghanistan and Sudan. The TI DALY age-standardized rates in 2015, were higher (3.1% of all DALYs) compared to 0.3% of the total number of DALYs from disease globally (Wang et al. 2016). Overall, there was a 9.9% increase from 1990 to 2015 for DALYs attributable to TI in EMR. From 1990 to 2015 DALY rates decreased in all countries except Pakistan and Libya, which increased 6.64% and 23.9%, respectively (Table 1; (Fig. 4). For all age groups and countries, YLLs were the primary contributor to DALYs (Fig. 4).

## Discussion

Our study is the first to report on the burden of TI in the EMR from 1990 to 2015. Our results show that EMR mortality rates due to TI have not fallen as quickly as the global estimates. Three countries—Libya, Pakistan, and Egypt—even have had increases in death rates. Our results show that TI are still a major health problem in the region and call for serious efforts to reduce their burden.

The YLL to YLD ratio can be used as an indicator for the severity of TI and the effectiveness of health system intervention. The higher this ratio is, the more severe and fatal the crashes are and the less effective interventions the health system provides are. This ratio may suggest that health care access and interventions are not up to global standards in the region, in spite of the economic growth and high SDI of some countries in the EMR. It has been shown that improved access to better-quality trauma care systems has played a role in the decreasing mortality rates due to TI in high-income countries (Noland 2003).

Most of the research on the impact of trauma care comes from high-income countries where systems have been implemented with few resource constraints. Assessments in LMICs have consistently identified enormous gaps in the resources needed to provide adequate care for the injured (Reynolds et al. 2017). A review describing reports that evaluated the impact of trauma care systems and system components in LMICs, identified reports from 32 countries. These reports, which describe potentially useful interventions to strengthen care for the injured in LMICs, were found in only about one-quarter of LMICs. The study suggests a substantial research gap that spans all regions (Reynolds et al. 2017). Another study suggested that mortality among people with life-threatening but potentially survivable injuries was sixfold lower in high-income countries (6%) than in low-income countries (36%) (Mock et al. 1993).

Globally, the burden of disease due to TI has decreased significantly since 1990, but this decrease is largely in high-income regions, with the reverse trend occurring in low-income and middle-income countries. Some studies have suggested that this is due to the growth in motorization and traffic density outpacing infrastructural development and levels of law enforcement (Ameratunga et al. 2006; Naghavi et al. 2009; WHO 2013). Countries with fast-growing economies have experienced rapid economic development that led to changes in lifestyle and environment and subsequently impacted health and mortality (Razzak et al. 2004; Luoma and Sivak 2012; Hyder and Vecino-Ortiz 2014). Motorization is rapidly increasing in the region (WHO 2015), and our study suggests that many regulations should be implemented. Safer roads, enforced traffic laws, formal driver education with more stringent driver license procedures and policies, and safe vehicle regulations need to be rigorously implemented to cope with the increase in access to vehicles, especially in high-income countries in the region. Countries with high numbers for specific causes, like pedestrian injuries in Oman, should implement specific measures to protect those at risk.

Similar to global trends (GBD 2015 Disease and Injury Incidence and Prevalence Collaborators 2016; Kassebaum et al. 2016; Wang et al. 2016), TI in the EMR disproportionately affect individuals who are in the economically productive age group of 15–44. This exerts an added pressure on the national economies of the EMR countries, especially those with limited resources (Mokdad et al. 2014, 2016 ). The burden of TI is significantly higher in males than females. This gender ratio is consistent with global trends (Wang et al. 2016). Besides being a public health burden, TI are also associated with an immense economic burden; it is estimated to cost EMR countries a total of US$7.5 billion per year, equivalent to 1–1.5% of the GDP of most countries in the region (Bishai et al. 2006).

Despite the continuous threat of the burden of injuries in the EMR, few studies have been conducted to assess the burden of TI in the region, with the available ones being limited to small-scale, city-based, or facility-based studies. Coverage of vital registration is low or absent in large parts of the EMR and issues of incompleteness and differences in death certification systems, definitions of variables, and methods of data collection usually compromise the quality of data (Setel et al. 2007; Mahapatra et al. 2007; Obermeyer et al. 2010; Joubert et al. 2012). In our study, it was necessary to predict estimates using models, relying on covariates and verbal autopsy data (Noland 2003; Kassebaum et al. 2016). We added police and mortuary data for TI to help predict level and age patterns in countries with sparse or absent cause of death data, even though we know from countries with near-complete vital registration data that police records tends to underestimate the true level of deaths. The large GBD mortality database allows us to use statistical models that can borrow strength when data is missing from similar countries, previous years, published literature if no raw data is available, published reports, police reports, media, etc. Although this ensures an estimate for all causes and all countries, estimates for populations and time periods with sparse or absent data are inherently less precise. While we attempt to capture all sources of uncertainty from sampling error, non-sampling error, and model specifications in the 95% uncertainty intervals, additional sources of uncertainty may not have been captured (Mathers et al. 2006; Byass et al. 2013).

A study in Saudi Arabia showed that rates of death from road traffic accidents based on police reports and on health registration data are different, and that unlike police-reported data, health registration does not show steadiness or decline in the rates of road traffic deaths (Barrimah et al. 2012). These inconsistencies may be caused by differences in definitions, or may reflect differences in data collection methods (Loo and Tsui 2007; Jeffrey et al. 2009), or road traffic officials may even be underreporting TI to avoid criticism from superiors who expect to see rates go down, as one study suggested (Dandona et al. 2008).

The fact that males were substantially more likely to die from TI than females may be correlated to the fact that in some of these countries less women drive motorized vehicles; also in most cases women are accompanied by men outside of their houses.

Suboptimal public awareness of the importance of the issue has resulted in diminished emphasis on road safety policies at the national level in EMR countries. Lack of solid, reliable data may be a significant barrier to policymakers’ prioritizing this major public health problem.

There are no definitive data on the number of people who survive with some form of permanent disability for every injury-related death, but estimates run between 10 and 50 times more permanent disabilities. As such, these injuries clearly contribute to the economic and social costs and have a negative impact on individuals, communities, and societies.

Many studies have shown that human behavioral factors collectively represent the main cause of three out of five road traffic crashes, and contribute to the cause in most remaining cases (Marshall et al. 1996; Evans 1996; Lyznicki et al. 1998; Sharma et al. 2002). A study in Saudi Arabia showed that more than 43% of unlicensed males drove a vehicle (El Bcheraoui et al. 2015). Among those male drivers (females are not allowed to drive by law), 86% engaged in at least one risky behavior while driving. Up to 95 and 98.5% of respondents reported not wearing a seat belt in the front (enforced by the law), and the back seat, respectively.

More attention must also be given to the needs of vulnerable road users, like pedestrians, children, and bicycle/motorcycle and public transport users. Making walking and cycling safer is critical to reducing the number of road traffic deaths and is important to promote non-motorized forms of transport.

### Conclusion

Our study highlights the significant burden of TI deaths and injuries in the EMR countries, and the need for improving trauma centers and implementation of a faster emergency care in the EMR. Strict monitoring and enforcement of traffic laws, and programs to increase awareness and proper education for drivers should be developed jointly by the Ministries of Health, Interior Affairs, and Education and provided through their channels.

## Electronic supplementary material

Below is the link to the electronic supplementary material.

Supplementary material 1 (XLSX 27 kb)
